# The process by which perceived autonomy support predicts motivation, intention, and behavior for seasonal influenza prevention in Hong Kong older adults

**DOI:** 10.1186/s12889-017-4608-x

**Published:** 2017-07-28

**Authors:** Pak-Kwong Chung, Chun-Qing Zhang, Jing-Dong Liu, Derwin King-Chung Chan, Gangyan Si, Martin S. Hagger

**Affiliations:** 1 Department of Physical Education, Hong Kong Baptist University, 224 Waterloo Road, Kowloon Tong, Kowloon, Hong Kong, Special Administrative Region of China; 2 Institute of Human Performance, University of Hong Kong, Pokfulam, Hong Kong, Special Administrative Region of China; 3 Sport Psychology Center, Hong Kong Sports Institute, Hong Kong, China; 4 Health Psychology and Behavioural Medicine Research Group, School of Psychology and Speech Pathology, Curtin University, Perth, Australia; 5 Department of Sport Sciences, University of Jyväskylä, Jyväskylä, Finland

**Keywords:** Elderly, Facemask wearing, Infection, Infectious diseases, Self-determination theory, Theory of planned behavior

## Abstract

**Background:**

This study examined the effectiveness of a theoretical framework that integrates self-determination theory (SDT) and the theory of planned behavior (TPB) in explaining the use of facemasks to prevent seasonal influenza among Hong Kong older adults.

**Methods:**

Data were collected at two time points in the winter in Hong Kong, during which influenza is most prevalent. At Time 1, older adults (*N* = 141) completed self-report measures of SDT (perceived autonomy support from senior center staff, autonomous motivation for influenza prevention) and TPB (attitude, subjective norm, perceived behavioral control, and intention for influenza prevention) constructs with respect to facemask used to prevent infection. Two weeks later, at Time 2, participants’ acceptance of a facemask to prevent influenza in the presence of an experimenter with flu-like symptoms was recorded.

**Results:**

Path analysis found that perceived autonomy support of senior center staff was positively and significantly linked to autonomous motivation for facemask use, which, in turn, was positively related to intentions to wear facemasks through the mediation of attitude, subjective norm, and perceived behavioral control. However, the effect of intention on facemask use was not significant.

**Conclusions:**

Results generally support the proposed framework and the findings of previous studies with respect to intention, but the non-significant intention-behavior relationship may warrant future research to examine the reasons for older adults not to wear facemasks to prevent seasonal influenza despite having positive intentions to do so.

## Background

Seasonal influenza is an acute epidemic of the influenza virus that quickly and easily spreads from person to person [1]. Annual seasonal epidemics peak during the winter months in temperate regions such as Hong Kong. Epidemics cause mild-to-severe illness and can be fatal in vulnerable groups such as the elderly [2]. Everyday preventive actions, such as facemask wearing, play an important role in the prevention seasonal influenza epidemics [3, 4]. This is because the main route of human-to-human transmission of the influenza virus is via respiratory droplets when an individual is close contact with someone who has the influenza virus and displays influenza symptoms [5]. In community settings, wearing a facemask is an effective means to minimize transmission of influenza, particularly in areas of high population density. In order to promote the adoption of behaviors like facemask use to prevent seasonal influenza infection, previous research has demonstrated that psychological factors such as motives and intentions are likely play a key role [6, 7].

Researchers have attempted to predict and understand health behavior using behavioral theories and models adopted from social psychology [8]. Among the models that have been applied to understand influenza prevention behaviors, most have adopted a social-cognitive approach, in which preventive behaviors such as vaccination and facemask wearing are viewed as intentional behaviors based on beliefs and expectancies [6, 9, 10]. Prominent among these social cognitive approaches is the theory of planned behavior (TPB) [11]. According to the theory, intention, defined as the extent to which individuals will invest effort in pursuit of an action, is the most proximal predictor of behavior. Intention is the function of attitude (i.e., personal evaluation of how good, useful, valuable, and pleasant the behavior is), subjective norm (i.e., beliefs that the behavior is consistent with the expectations of significant others), and perceived behavioral control (i.e., beliefs in the availability of sufficient personal resources, in relation to barriers or risk factors, to execute the behavior). The TPB has been widely applied and tested in health-related behavioral contexts with meta-analytic studies supporting its effectiveness in predicting various health behaviors [12, 13]. The predictive efficacy of the TPB with respect to influenza prevention has also been supported in research on influenza vaccination behaviors [14, 15].

Apart from the TPB, another important theory in social psychology that has been frequently applied in health contexts is self-determination theory (SDT) [16, 17]. SDT makes the distinction between different forms of motivation based on its *quality* rather than *quantity*: autonomous motivation, controlled motivation, and amotivation. Autonomous motivation is the most self-determined form of motivation. Autonomously motivated individuals perform behaviors for the intrinsic value of the behavior, or to attain personally-important values or goals that represent their true sense of self. In contrast, individuals experiencing actions as controlled motivated engage in behaviors due to external pressures or externally-referenced obligations. Amotivation represents a lack of motivation such that individuals do not know why they engage in their actions at all. Individuals experiencing actions as controlled motivated will only perform an action when the external demands or controlling contingencies are present and the actions will cease once the contingencies are no longer present. Of the three types of motivation identified in SDT, autonomous motivation is proposed to be more favorable to behavioral persistence and well-being because the individual is motivated for self-referenced reasons and in the absence of external reinforcement or contingency [17].

Consistent with the need to promote autonomous motivation toward health behaviors, according to the SDT, creating a context or ‘environment’ that cultivates autonomous motivation is an effective means to promote autonomous motivation and change behavior [18, 19]. In an influenza prevention context, interventionists may capitalize on autonomy support techniques as a means to promote autonomous motivation [20]. For community-dwelling older adults, their senior center serves as an important vehicle to present autonomy-supportive messages to promote behaviors that could enhance health and well-being, including preventive behaviors to reduce the transmission of potentially life-threatening infections [21].

Recent approaches have attempted to integrate concepts from the TPB and SDT in a unified model to explain health behavior. The integrated model proposes that the motivational variables from SDT are distal predictors of the social cognitive variables from TPB, which are, in turn, considered the proximal antecedents of action [22, 23]. The rationale for the integration arises from a key tenet of SDT that individuals who perceive their reasons for acting autonomously should view their behavior as one they should approach. In order to enact the behavior, the individual should align his or her beliefs regarding future engagement in the behavior to be consistent with their motives (i.e., to form positive beliefs about the behavior and form intentions to engage in it). Promoting autonomous motivation, therefore, may be a means to promote behavioral engagement through the enhancement of positive beliefs regarding the behavior and the formation of intentions to engage in the behavior in the future. The model has been tested in many health contexts including physical activity, binge drinking, injury management, sugar consumption, and myopia prevention [24–29].

Although a recent study also applied the integrated model to predict behavior in the context of H1N1 pandemic, participants were presented with a hypothetical situation and were not actually facing an actual pandemic and no actual facemask wearing behavior was assessed. [20]. Even though intention is theoretically the most proximal predictor of behavior, there has been growing number of concerns about the differential predictive power of intention on health behavior, and the so-called intention-behavior gap has raised concerns over the adequacy of intentions in explaining future behavior [30–32]. It is, therefore, important that test of the integrated model include prospective measures of actual behavior, particularly in influenza prevention contexts.

The purpose of the current study was to examine the efficacy of an integrated model based on SDT and TPB to predict facemask wearing to prevent seasonal influenza in a sample of Hong Kong older adults. We adopted a two-week prospective correlational design during Hong Kong’s winter, the peak season for seasonal influenza, and we included an actual measure of the target behavior. Specifically, we measured the motivational and social cognitive variables from the integrated model at Time 1 (baseline) and followed that up two-weeks later at Time 2 with an assessment of actual facemask-wearing behavior. Building on the integrated model, it was hypothesized that: (1) Perceived autonomy support from senior center staff for facemask wearing would be positively and directly related to autonomous motivation (hypothesis 1a) and positively and indirectly related to attitude, subjective norm, and perceived behavioral control via the mediation of autonomous motivation (hypothesis 1b); (2) Autonomous motivation would be positively and directly related to attitude, subjective norm, and perceived behavioral control (hypothesis 2a) and positively and indirectly related to intention via the mediation of attitude, subjective norm, and perceived behavioral control (hypothesis 2b); (3) Attitude, subjective norm, and perceived behavioral control would be positively and directly associated with intention (hypothesis 3a) and positively and indirectly related to actual facemask wearing behavior via the mediation of intention (hypothesis 3b).

## Method

### Participants

Ethical clearance was granted by the committee of Research Ethics and Safety (HASC) at Hong Kong Baptist University. We obtained approval and on-site assistance to recruit participants from one senior center in Hong Kong using convenience sampling. This center has over 1500 registered Chinese-speaking community-dwelling Hong Kong older adults aged 60 years and older. A total of 180 older adults expressed an interest in participating to senior center staff and members of the research team. Of these eligible participants, 141 agreed to participate and signed consent forms (response rate = 78.33%). The sample comprised of 14 males and 127 females (*M* age = 75.23, *SD* = 6.52, range 60 to 85) participated the study at Time 1. At Time 2, 137 participants (13 males, 124 females; *M*_*age*_ = 75.10, *SD* = 6.49) remained in the study (retention rate = 97.16%).

### Design and procedure

Data were collected at two time points with a two-week interval in the November of 2014. At Time 1, during the peak season of winter influenza in Hong Kong, participants completed survey measures of study variables under the supervision of researchers following an introductory session in which the purpose and procedure of the study was explained and written informed consent obtained. The surveys took 10 to 15 min to complete. Senior center staff were not present during the completion and were not able to see participants’ responses. At Time 2, participants were invited to a face-to-face interview in a private room to ostensibly assess their knowledge of H1N1. The true purpose of the interview was to assess participants’ facemask wearing behavior for influenza prevention. During the course of the interview, the interviewer wore a facemask and feigned influenza-like symptoms (e.g., coughing, sneezing). Before the start of the interview, the interviewer told the participants in an offhand manner that he/she had caught “the ‘flu”, and that surgical facemasks were available at the desk that they could take and use for free. This allowed the interviewers to record participants’ facemask-wearing behavior. On completion of the interview, participants were debriefed regarding the cover story and informed that the interviewer had feigned influenza symptoms and worn the facemask as a prop to model the behavior.

### Measures

#### Perceived autonomy support

Perceived autonomy support from staff of senior center was measured using the six-item Health Care Climate Questionnaire (HCCQ) [18]. Participants were provided with a common stem: “When the staff at the senior center asks me to wear a facemask in an enclosed public place…” followed by the six items (e.g., “I feel that he/she has provided me choices and options”). Responses were made on a 7-point scale anchored by 1 (“not at all true”) and 7 (“very true”). The Chinese version of the HCCQ has demonstrated sufficient validity and reliability [20].

#### Autonomous motivation

The six-item autonomous motivation subscale from the Treatment Self-Regulation Questionnaire (TSRQ) [33] was used to measure autonomous motivation. The stem of TSRQ items was modified to refer the specific behavior of interest (i.e., “I want to wear a facemask in an enclosed public place because …”). Participants responded to items on 7-point Likert scales with 1 (“not at all true”) and7 (“very true”) as scale anchors. The Chinese version of TSRQ has demonstrated sufficient validity and reliability [20].

#### Theory of planned behavior

Measure of the TPB variables were based on Ajzen’s guidelines [34]. Measures of subjective norm (3 items; e.g., “It is expected of me to wear facemask in an enclosed public place in the forthcoming month.”), perceived behavioral control (5 items; e.g., “It is possible for me to wear facemask in an enclosed public place in the forthcoming month.”), and intention (3 items; e.g., “I intend to wear facemask in an enclosed public place in the forthcoming month.”) were rated on 7-point Likert scales with 1 (“strongly disagree”) and 7 (“strongly agree”) as anchors. Attitudes were measured using 7 items preceded by a common stem, “For me to wear facemask in an enclosed public place in the forthcoming month would be …” followed by a series of 7-point semantic differential scales: extremely harmful-extremely beneficial, extremely unpleasant-extremely pleasant, extremely worthless-extremely valuable, extremely bad-extremely good, and extremely unenjoyable-extremely enjoyable.

#### Facemask-wearing behavior

Facemask-wearing behavior was assessed at Time 2 during the face-to-face interview. The interviewer disclosed in an offhand manner that they had caught influenza and feigned influenza symptoms. Participants were made fully aware of the availability of facemasks and that they were free to use. The interviewer was trained to observe and record the correct use of the facemasks according to World Health Organization guidelines (i.e., complete coverage of mouth and nose) [5]. The interviewers recorded a 1 (“yes”) when participants took and used the facemasks correctly and otherwise recorded a 0 (“no”).

#### Control variables

Control variables included past facemask-wearing habit (i.e., whether or not they had worn facemasks previously; 1 item), knowledge of the benefits of facemask wearing (i.e., knowing that facemask wearing can prevent influenza; 1 item), frequency of influenza infection during the past 6 months (1 item), and perceived susceptibility. Perceived susceptibility (e.g., “I have an increased risk of falling ill with influenza”; 3 items) was rated on a 7-point Likert scale with 1 (“strongly disagree”) and 7 (“strongly agree”) as scale anchors [7].

### Data analysis

**Fig. 1:**
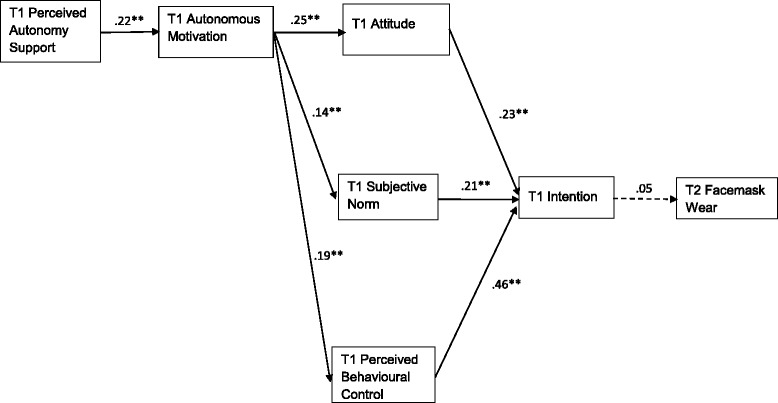
Path Estimates of the Integrated Model. For clarity reasons, the statistically non-significant paths are not displayed. ** *p* < .01; * *p* < .05

Given that the Time 2 facemask wearing behavior was a categorical variable (yes/no), the proposed integrative model (see Fig. 1) was tested using path analysis (i.e., observed variables) with a variance-adjusted weighted-least squares (WLSMV) estimation method using the Mplus 7.3 [35]. Variables including facemask wearing habit, knowledge of facemask wearing benefits, frequency of influenza during the past 6 months, and perceived susceptibility were included as control variables. Adequacy of the fit of the proposed model with the data was based on multiple criteria for assessing goodness of fit including the comparative fit index (CFI), the root-mean-square error of approximation (RMSEA) and the weighted root mean square residual (WRMSR). Values exceeding .90 for the CFI and less than .08 and 1.00 for the RMSEA and WRMSR [36, 37], respectively, indicate good fit. For our tests of mediated effects, mediation was confirmed when the indirect effect of a predictor variable (e.g., autonomous motivation) on an outcome variable (e.g., intention) via a mediator (e.g., attitude) was statistically significant and the confidence interval of the effect size did not include zero.

## Results

### Preliminary analysis

In terms of the facemask wearing habit, most of the participants reported wearing facemasks previously (*n* = 124), while a small number of them reported that they did not been used to wearing facemasks previously (*n* = 17). With respect to knowledge of the benefits of facemask wearing, most of the participants reported knowing facemask wearing can prevent flu (*n* = 132), while a small number reported they did not know facemask wearing can prevent flu (*n* = 9). With regards to the frequency of influenza during the past 6 months, most of the participants reported that they had not caught influenza (*n* = 120). In comparison, only 11 participants reported that they caught influenza once, seven participants reported they had had the flu twice, and only three participants reported catching influenza three times. Missing data analysis revealed no significant pattern (missing data = 0.18%) and the small number if missing cases was replaced using mean substitution. Descriptive statistics and intercorrelations of the study variables are presented in Table 1.

**Table 1:** Summary of correlations, means, standard deviations (SD), and internal consistencies of the self-reported questionnaires

		Range	Mean	*SD*	*α*	1	2	3	4	5
1	Perceived autonomy support	6–42	33.36	4.27	.77					
2	Autonomous motivation	6–42	36.02	2.45	.67	.26^**^				
3	Attitude	7–49	38.63	5.97	.68	.25^**^	.33^**^			
4	Subjective norm	3–21	16.55	3.10	.79	.17^*^	.18^**^	.34^**^		
5	Perceived behavioral control	5–35	29.94	2.97	.90	.28^**^	.24^**^	.40^**^	.23^**^	
6	Intention	3–21	16.56	3.30	.88	.11	.22^*^	.45^**^	.39^**^	.57^**^

^**^ *p* < .01 at two-tailed

^*^ *p* < .05 at two-tailed

### Path analysis

The model exhibited adequate fit with the data, *χ* ^*2*^ (3) = 1.58, *p* = .664, CFI = 1.00, WRMR = .178, RMSEA (90% *CI*) = .000 (.000, .112). Direct, indirect, and total effects of the path estimates of the integrated model are presented in Table 2 and Fig. 1. Effects of the control variables were not significant except the effect of frequency of influenza over the previous six months on autonomous motivation (β = −.18, 95%*CI* [−.344 to −.024], *p* = .025), and they are not, therefore, displayed in Fig. 1.

**Table 2:**
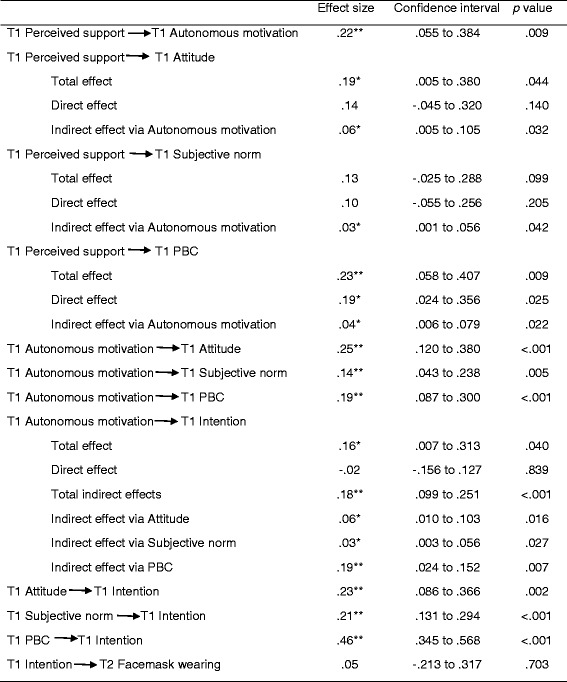
Total, direct, and indirect effects of the proposed paths in the Integrated Model

Focusing on the effects encompassed by hypothesis 1, we observed a statistically significant and positive effect of perceived autonomy support on autonomous motivation consistent with our hypothesis (hypothesis 1a). The indirect effects of perceived autonomy support on attitude and subjective norm were statistically significant as hypothesized (hypothesis 1b), although the direct effects were not. As predicted, the total, direct, and indirect effects of perceived autonomy support on perceived behavioral control were also statistically significant (hypothesis 1b).

Statistically significant and positive associations were observed for autonomous motivation on attitude, subjective norm, and perceived behavioral control, as hypothesized (hypothesis 2a). The indirect effects of autonomous motivation on intention mediated by attitude, subjective norm, and perceived behavior control were all statistically significant, consistent with predictions (hypothesis 2b).

We found statistically significant and positive effects of attitude, subjective norm, and perceived behavioral control on intention, as predicted (hypothesis 3a). However, contrary to predictions, the effect of intention on our measure of facemask wearing was small and not statistically significant (hypothesis 3b).

## Discussion

Building on an integrated model of SDT and TPB [22, 23, 27], we tested effects among perceived autonomy support, autonomous motivation, attitudes, subjective norms, perceived behavioral control, intention, and behavior for facemask wearing during peak influenza season among elderly people in Hong Kong. Consistent with our hypotheses, we found statistically significant and positive effects of (1) perceived autonomy support on autonomous motivation, attitude, subjective norm, and perceived behavior control; (2) autonomous motivation on attitude, subjective norm, and perceived behavioral control, and intention; and (3) attitude, subjective norm, and perceived behavioral control, on intention. There were also significant and positive indirect effects of perceived autonomy support on attitude, subjective norm, and perceived behavior control via autonomous motivation, and of autonomous motivation on intentions via attitude, subjective norm, and perceived behavior control. However, we found no effect of intention on our measure of actual facemask wearing, and, consequently, no indirect effects of the motivational and social-cognitive variables on behavior mediated by intention. Our findings, therefore, suggest that the motivational and social-cognitive constructs outlined in the integrated model are effective in predicting intentions to wear a facemask during the peak winter season, but, critically, intentions were not predictive of individuals’ actual engagement in facemask wearing behavior.

### Efficacy of the integrated model and its component theories

Current findings are consistent with the hypotheses of the integrated model and its component theories when it comes to predicting intentions. Perceived autonomy support was related to autonomous motivation for adopting health-related behaviors consistent with the predictions of SDT [16, 17, 38], a key component theory of the integrated model [22, 23]. The finding is also consistent with the findings of a previous study on facemask wearing [20]. The results suggest that establishing an autonomy supportive environment or ‘motivational climate’ toward influenza prevention behaviors such as facemask wearing will be effective in fostering autonomous motivation toward the behavior.

Consistent with previous studies of influenza prevention behaviors such as facemask wearing and vaccination using the TPB [14, 15, 20], attitudes, subjective norms, and perceived behavior control had positive, significant, and medium-to-large associations with the intentions of wearing facemasks in Hong Kong older adults. Among these three variables, perceived behavioral control had the strongest effects relative to attitudes and subjective norms. It was also the most prominent variable in the transmission of indirect effects of autonomous motivation on intention. Given that the TPB is, by and large, an integration of the personal (i.e., attitude) and social (i.e., subjective norm) antecedents of intention from the reasoned action approach [39, 40], and the self-efficacy related component from social cognitive theory [41], the increase of older adults’ personal resources (e.g., self-efficacy) and the decrease of the hindrance from barriers (e.g., easy access to facemasks when needed) seem to be most efficacious in accounting for variance in intentions to engage in the focal health behavior. Current findings provide formative evidence to support interventions to facilitate greater confidence in wearing facemasks and to overcome barriers to doing so.

The significant and positive direct effects of perceived autonomy support on attitude and perceived behavioral control indicated that a supportive motivational climate may affect the belief-based TPB variables. This is in line with previous studies [20, 24, 25, 27] and it further corroborates the integrated model [22, 23]. That is, autonomous motivation from SDT underpins the belief-based social cognitive variables of TPB, and the current study extends this to facemask wearing to prevent influenza transmission. The prominent role of control-related beliefs for this health behavior, and its mediating role in translating autonomous motives into intentions, is consistent with other theories which feature self-efficacy as a prominent component [42]. It is also consistent with previous research that has adopted the integrated model and demonstrated that perceived behavioral control is a key predictor and mediating factor of the influence of autonomous motivation on intentions [43].

### Intentions do not predict behavior

The intentions of facemask wearing alone may be insufficient or unimportant when it comes to engaging in this particular behavior. In a post hoc analysis, we found that the majority of participants (89.05%) scored above the mid-point on the intention scale but only two fifths of them chose to wear the face masks (39.40%) in our scenario. Considering these data, it would be reasonable to assume that the majority of participants should be considered inclined abstainers or unsuccessful intenders [30, 44], or have weak or unstable [45–47] intentions. Given that older adults’ facemask wearing habits, knowledge of whether wearing facemasks can prevent influenza, frequency of influenza over the previous 6 months, and perceived susceptibility to influenza were controlled for, it is reasonable to conclude that the current findings are consistent with the well-documented intention-behavior gap.

A number of factors may account for this ‘gap’. First, it emerged from the qualitative debriefing session conducted after the study had been completed that many of the participants chose not to wear a facemask because the interviewer was wearing a facemask, and they believed that they were, therefore, safe. Others reported that they were put off wearing facemasks themselves because it would make it difficult or uncomfortable to breathe, a notable barrier. As this is anecdotal evidence rather than a formal assessment, it may be important in future research to examine participants’ specific beliefs regarding the behavior, rather than global or direct measures of attitudes and perceived behavioral control. Such an approach may identify the influential behavioral (e.g., beliefs that people with illnesses will wear facemasks to reduce risk to others) and control (e.g., wearing masks makes it difficult or uncomfortable to breathe) beliefs that may influence the formation of intentions. Omission the beliefs that precede intentions aside, it may also be important to study the underlying volitional mechanisms from dual-phase models of action like the model of action phases [48] and the Health Action Process Approach [44] that determine the enactment of intentions. Incorporation of volitional components like implementation intentions, action planning, and coping planning into the study of facemask wearing may assist in identifying the possible moderators of the intention behavior relation.

Similarly, it may also be important to look at the implicit, non-conscious or ‘automatic’ influences on action. Research has indicated that self-reported habit predicts behavior as do implicitly measured attitudes and motives [49–51]. Such influences likely account for action independent of the intentional route to behavior, and therefore determines action beyond an individual’s awareness. The likely process involved is that the implicit beliefs are associated with schema, or memory structures, which outline patterns of action which have been well learned and reinforced over time. Such patterns are activated when the implicit belief or attitude is cued, and, as a consequence, an individual’s behavior is initiated automatically [27, 52]. This does not mean that individuals act like ‘automatons’, blindly carrying out actions. The actual behavior may involve considerable planning and effort, it is just that the behavioral pattern is set in motion in a quick, efficient manner making the behavior subjectively very easy to enact. Implicit beliefs are also more likely to facilitate the enactment of the intended action, or failing to do the action, if it is either rewarding or relatively easy to do. Future research should examine individuals’ implicit beliefs toward illness and implicit beliefs toward the behavior, with respect to facemask wearing and assess their contribution to action. This would be consistent with further integrated models that have attempted to incorporate implicit components alongside motivational and volitional components of action in a unified framework [23].

### Strengths and limitations

The current study has numerous strengths including a focus on an important health-related behavior aimed at preventing influenza infection in a vulnerable population and has the potential to save lives, recruitment of a hard-to-reach population of older adults, adoption of an integrated theoretical model that has shown efficacy in predicting health behavior, and the use of valid and reliably instruments and a prospective design, and the use of an externally-verified observational measure of behavior instead of self-reports which have inherent biases.

Although our findings offer insight into the motivational processes that relate to facemask use in Hong Kong older adults to prevent influenza transmission during peak season, a number of limitations of the study should be identified and discussed. First, participants in the current study were ethnically and geographically homogeneous since they were recruited from the same senior center. All participants were Hong Kong Chinese, and most of them were female. In future, it would be important to replicate the findings in more diverse sample of older adults to provide further evidence on the validity and generalizability of study findings. Secondly, inference of causal relations among variables in the integrated model cannot be made even with the adoption of a prospective design. The causal direction of effects in the model can only be inferred from the theory rather than the data [53], it is, therefore, imperative that researchers also adopt experimental paradigms to manipulate key variables in the nomological network proposed by the theory and observe their effects on outcomes. Finally, the current study did not conduct an a priori statistical power analysis and adopted a manifest rather than latent variable approach given the relatively small sample. Future research should seek to conduct a power analysis beforehand, collect data from a larger sample and use a latent variable modeling approach which would control for measurement error [54].

## Conclusion

In conclusion, findings of the current study provided support for the motivational aspects of the integrated model to the wearing of facemasks to prevent seasonal influenza in a sample Hong Kong older adults. However, our research did not support the link between intentions to wear facemasks and a situated decisional measure of facemask wearing in a real-life context. Further examination of the potential alternative processes and moderators of the link between intentions and behavior in this context may provide insight on contexts in which intentions to wear facemasks lead to actual facemask wearing behavior, and the processes involved.

## Abbreviations

CFI: Comparative fit index
HCCQ: Health Care Climate Questionnaire
RMSEA: Root-mean-square error of approximation
SDT: Self-determination theory
TPB: Theory of planned behavior
TSRQ: Treatment Self-Regulation Questionnaire
WLSMV: Variance-adjusted weighted-least squares
WRMR: Weighted root mean square residual
